# Artificial intelligence–based ECG as a triage tool for acute myocardial infarction: a diagnostic systematic review and meta-analysis

**DOI:** 10.1093/ehjdh/ztag075

**Published:** 2026-08-29

**Authors:** Giulia Caldeira Gaelzer, Pedro Gomes Batista, Marcela Vasconcelos Montenegro, Railla Raquel Albino dos Santos Silva, Mushrin Malik, Maria Leticia Carnielli Tebet, Caroline O Fischer-Bacca, Juliana Giorgi, Gustavo Lenci Marques

**Affiliations:** Pontifical Catholic University of Paraná, Curitiba, Paraná, Brazil; Federal University of Paraíba, João Pessoa, Paraíba, Brazil; University of Pernambuco, Recife, Pernambuco, Brazil; Federal University of Ceará, Fortaleza, Ceará, Brazil; Beth Israel Deaconess Medical Center, Boston, MA, USA; Pontifical Catholic University of Paraná, Curitiba, Paraná, Brazil; University Center for the Development of Alto Vale, Rio do Sul, Santa Catarina, Brazil; Hospital Sírio-Libanês, São Paulo, SP, Brazil; Hospital Israelita Albert Einstein, São Paulo, SP, Brazil; Pontifical Catholic University of Paraná, Curitiba, Paraná, Brazil; Federal University of Paraná, Curitiba, Paraná, Brazil

**Keywords:** Artificial intelligence, Electrocardiography, Acute myocardial infarction, Acute coronary syndromes, Diagnostic accuracy, Triage

## Abstract

Early identification of acute myocardial infarction (AMI) remains challenging, particularly in non-ST-segment elevation presentations and occluded myocardial infarction, where conventional electrocardiogram (ECG) interpretation has limited sensitivity. Artificial intelligence–enabled ECG (AI-ECG) has emerged as a promising strategy to enhance early triage and diagnostic accuracy. To systematically evaluate the diagnostic performance of AI-enabled ECG algorithms for the detection of AMI, including ST-segment elevation myocardial infarction (STEMI) and non-ST-segment elevation myocardial infarction (NSTEMI), across diverse clinical settings. This diagnostic systematic review and meta-analysis was conducted in accordance with PRISMA guidelines and registered in PROSPERO (CRD420261292271). PubMed, Embase, and Cochrane CENTRAL were searched through January 2026. Studies evaluating AI-based ECG models for AMI detection and reporting sufficient data to reconstruct 2 × 2 contingency tables were included. Pooled sensitivity, specificity, positive predictive value (PPV), and negative predictive value (NPV) were estimated using random-effects models (restricted maximum likelihood). Summary receiver operating characteristic (SROC) curves and area under the curve (AUC) were generated. Pre-specified subgroup analyses were performed for STEMI and NSTEMI Ten observational studies comprising 94 510 participants were included. For overall AMI detection,. AI-ECG demonstrated a pooled sensitivity of 89.4% (95% CI, 79.7–94.8) and specificity of 96% (95% CI, 91.2–98.2). The pooled NPV was 98.7% (95% CI, 94.1–99.7), and the pooled PPV was 73.3% (95% CI, 50.2–88.2). The SROC AUC was 0.97 (95% CI, 0.92–0.98). In STEMI, pooled sensitivity and specificity were 94.4% and 97.5%, respectively (AUC 0.98). In NSTEMI, pooled sensitivity was lower at 65.0%, with specificity of 87.5% and an AUC of 0.71. Heterogeneity was substantial, particularly among NSTEMI cohorts. AI-enabled ECG demonstrates high sensitivity and consistently excellent negative predictive value for AMI detection, supporting its role as a scalable, non-invasive triage adjunct at first medical contact. These findings highlight the potential of AI-ECG to facilitate early rule-out strategies and improve prioritization of patients requiring urgent ischaemic evaluation. Beyond diagnostic accuracy, AI-ECG may support probabilistic risk stratification and integration into early clinical decision-making pathways.

## Graphical Abstract

**Graphical Abstract:**
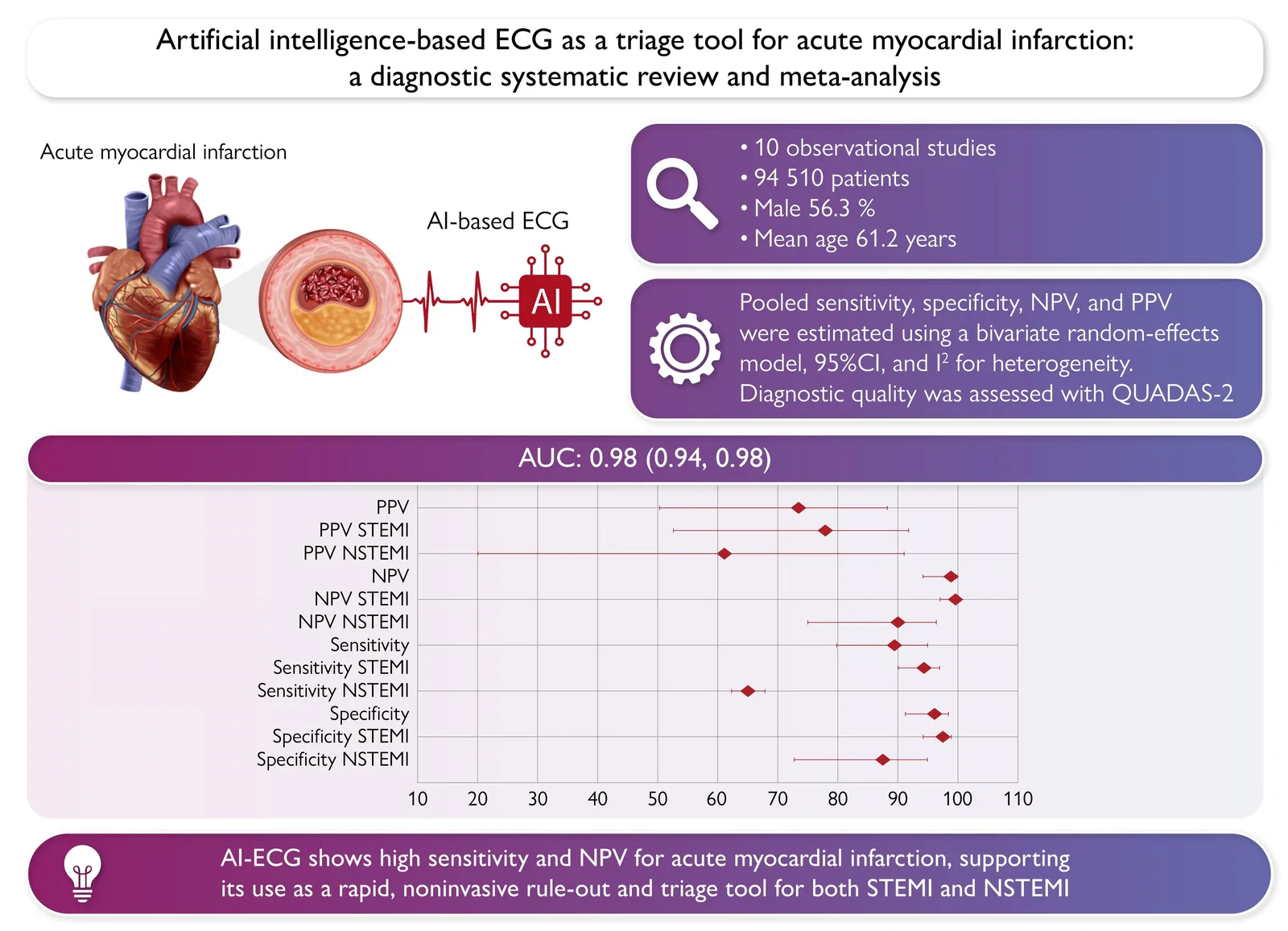

## Introduction

Rapid and accurate identification of acute coronary syndromes (ACS), particularly acute myocardial infarction (AMI), remains a cornerstone of contemporary cardiovascular care.^1^ The 12-lead electrocardiogram (ECG) is universally available and central to early triage, yet conventional rule-based ECG interpretation lacks sufficient sensitivity, especially in non-ST-segment elevation presentations and in occluded myocardial infarction (OMI), where timely reperfusion is frequently delayed. Misclassification at first medical contact continues to contribute to preventable morbidity, mortality, and inefficient use of catheterization laboratory resources.^2,3^

Recent advances in artificial intelligence (AI) and deep learning have transformed ECG analysis, enabling the extraction of complex temporal, spatial patterns that are imperceptible to human readers and traditional algorithms.^4,5^ Across diverse clinical settings, including emergency departments, prehospital systems, and telemedicine networks, AI-enabled ECG models have consistently demonstrated superior diagnostic accuracy for STEMI, NSTEMI, and OMI compared with conventional ECG criteria and, in some cases, expert interpretation. Importantly, several studies highlight a high negative predictive value (NPV), supporting the role of AI-ECG as an effective rule-out and triage tool for suspected AMI.^6,7^

Despite the rapid growth of this field, the existing evidence is heterogeneous with respect to model architecture, ECG acquisition (single-lead vs. 12-lead), clinical context (prehospital vs. in-hospital), reference standards, and outcome definitions. Individual studies are often limited by single-centre design, modest sample sizes, or a focus on specific AMI phenotypes, thereby limiting generalizability and precluding precise estimates of diagnostic performance across care pathways. Moreover, the extent to which AI-ECG performance is consistent across contemporary real-world cohorts has not been systematically quantified.

Accordingly, we performed a pooled analysis of 10 studies evaluating AI-enabled ECG algorithms for the detection of AMI and related high-risk ischaemic phenotypes. By synthesizing available evidence across multiple clinical environments and AI approaches, this study aims to provide a robust, quantitative assessment of diagnostic performance, to compare diagnostic accuracy across AMI subtypes, and to clarify the potential role of AI-ECG as a scalable decision-support tool in early ACS triage and management.

## Methods

This analysis was prospectively registered on the International Prospective Register of Systematic Reviews in Health and Social Care (PROSPERO: Number CRD420261292271). The study was designed and conducted in accordance with the Cochrane Collaboration Handbook for Systematic Reviews of Interventions^8^ and the Preferred Reporting Items for Systematic Reviews and Meta-Analyses (PRISMA) Statement guidelines.^9^ Ethical approval and informed consent were not required because the analysis was based exclusively on data from previously published studies, all of which had received approval from relevant ethics committees or institutional review boards.

### Study eligibility

Inclusion in this meta-analysis was restricted to studies meeting all of the following criteria:

1. Evaluated artificial intelligence–based or machine learning–based ECG algorithms as an index test for the detection of acute myocardial infarction (AMI);
2. Included adult patients (≥18 years) presenting with suspected ACS in any clinical setting (prehospital, emergency department, or in-hospital);
3. Used an appropriate reference standard for AMI diagnosis, including clinical adjudication based on established diagnostic criteria, cardiac biomarkers, and/or coronary angiography; and
4. Reported sufficient data to allow reconstruction of 2 × 2 diagnostic contingency tables, including true positives, false positives, false negatives, and true negatives. Studies were excluded if they:
  1. Did not evaluate diagnostic accuracy for AMI detection;
  2. Focused exclusively on prognostic or risk prediction outcomes;
  3. Enrolled paediatric populations;
  4. Were non-original research articles, including conference abstracts, editorials, narrative reviews, case reports, or preprints; or
  5. Lacked sufficient data to derive diagnostic accuracy estimates.

### Search strategy and data extraction

We systematically searched MEDLINE, Embase, and the Cochrane Central Register of Controlled Trials for studies published in English from database inception to January, 2026. The full electronic search strategy for each database is provided in Supplementary material online, *Methods S1*. In addition, reference lists of included studies, as well as relevant systematic reviews and meta-analyses, were manually screened to identify additional eligible studies. Title/abstract and full-text screening were independently performed by two reviewers (G.G. and M.L.C.T.), with disagreements resolved by a third author (G.L.M).

Data were independently extracted by two investigators (G.G. and P.G.B.) using predefined criteria. A standardized data extraction form was used to collect study-level characteristics (first author, year of publication, study design, follow-up duration, and sample size) and baseline population characteristics, including sex distribution, age, and the prevalence of hypertension, diabetes mellitus, hyperlipidaemia, and coronary artery disease.

In the included studies, AI-ECG models generally produced continuous probability scores for AMI detection. Diagnostic thresholds used to derive sensitivity and specificity were defined according to each study’s validation strategy, most commonly based on receiver operating characteristic (ROC) analysis or probability cutoffs reported by the original investigators.

### Statistical analysis

For each included study, 2 × 2 contingency tables were constructed to derive sensitivity, specificity, positive predictive value (PPV), and NPV. Pooled estimates of diagnostic accuracy were obtained using random-effects models to account for between-study heterogeneity. Random-effects models were fitted using restricted maximum likelihood estimation. Statistical heterogeneity was quantified using the *I*^2^ statistic. Overall test performance was summarized with a summary receiver operating characteristic (SROC) curve. Global diagnostic accuracy was assessed by estimating the area under the curve (AUC), with uncertainty evaluated using parametric bootstrapping. Pre-specified subgroup analyses were performed according to MI subtype, including STEMI and NSTEMI. Additional subgroup analyses were performed to explore potential sources of heterogeneity across studies, including differences in study design (prospective vs. retrospective). Sensitivity analyses were conducted using a leave-one-out approach, in which each study was sequentially excluded and pooled estimates of sensitivity, specificity, PPV, and NPV were recalculated using random-effects models. This procedure assessed the robustness of the meta-analytic findings and the influence of individual studies on the overall summary estimates. Results were presented using forest plots and graphical summaries. All statistical analyses were performed with R software, version 4.5.0 (R Foundation for Statistical Computing, Vienna, Austria).

### Risk of bias assessment and publication bias assessment

The risk of bias of the included studies was assessed using the Quality Assessment of Diagnostic Accuracy Studies–2 (QUADAS-2) tool.^10^ Two reviewers (M.V.M. and R.R.A.S.) independently evaluated all diagnostic accuracy studies, and any discrepancies were resolved by consensus. The QUADAS-2 tool assesses methodological quality across four domains: patient selection, index test, reference standard, and flow and timing. Potential publication bias was evaluated using Deeks’ funnel plot asymmetry test, with *P* < 0.10 considered indicative of significant asymmetry.

## Results

### Study selection and characteristics

**Figure 1:**
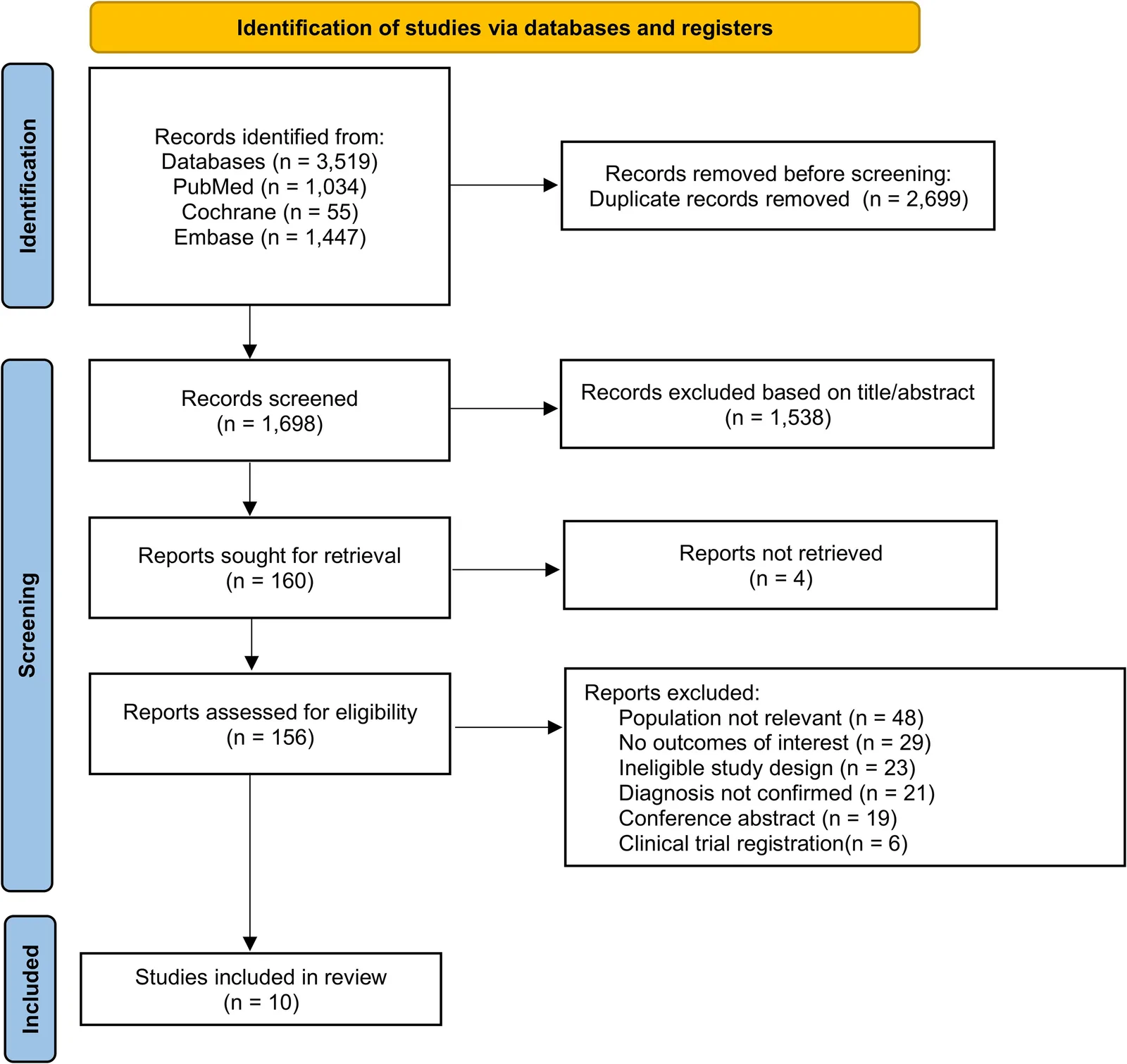
Preferred reporting items for systematic reviews and meta-analyses flow diagram of study screening and selection.

The literature search identified 3519 records, of which 2699 duplicates were removed, leaving 1698 records for title and abstract screening. After screening, 1538 records were excluded as not relevant, and 156 full-text articles were assessed for eligibility. Ten studies^6,7,11–18^ met the inclusion criteria (*Figure 1*). The primary reasons for full-text exclusion were non-relevant population, absence of outcomes of interest, ineligible study design, unconfirmed diagnosis, conference abstracts, and clinical trial registrations.

The 10 included observational studies comprised 94 510 participants from North America, East Asia, and Latin America. Most were retrospective, with sample sizes ranging from 217 to 35 981 and mean or median ages between 59 and 65 years; the overall proportion of male participants was 53.68%. The pooled prevalence of hypertension, diabetes mellitus, hyperlipidaemia, and coronary artery disease were 41.94%, 22.98%, 27.51%, and 23.49%, respectively. All studies evaluated AI–based ECG models for AMI detection, most commonly deep neural networks and convolutional neural networks, including ResNet-based, CNN–LSTM hybrid, and proprietary systems (PMCardio and AiTiAMI). Study-level characteristics are provided in *Table 1*.

**Table 1:** Baseline characteristics of included studies

Study	Number of participants	Study design	Country	Male, *n* (%)	Follow-up, months (Mean or Median)	Age, years (Mean or Median)	AI/DL used	HTN, *n* (%)	Diabetes, *n* (%)	HLD, *n* (%)	CAD, *n* (%)
AERO-ACS Choi *et al*.^12^	217	Single-centre retrospective cohort study	USA	130 (59.9)	12	65.1	PMCardio AI ECG model	NR	NR	NR	NR
Al-Zaiti *et al*.^13^	1244	Prospective observational cohort study (EMPIRE study)	USA	684 (55.0)	1	59	Machine-learning classifiers on 12-lead ECG	848 (68.2)	328 (26.4)	NR	427 (34.3)
Chang *et al*.^14^	35 981	Retrospective observational study	Taiwan	20 149 (56)	NR	65.06 ± 17.9	Long short-term memory (LSTM) deep learning model	NR	NR	NR	NR
Chen *et al*.^15^	275	Prospective observational cohort study	Taiwan	NR	NR	NR	CNN-LSTM deep learning model	NR	NR	NR	NR
ECG-SMART-NET Riek *et al*.^16^	7397	Multisite retrospective observational cohort	USA	4005 (54)	NR	59 ± 16	CNN (modified ResNet-18: ECG-SMART-NET)	2870 (39)	1401 (19)	1882 (25)	1375 (19)
Liu *et al*.^17^	30 761	Single-centre retrospective case–control study	Taiwan	15 718 (51.1)	NR	60.9–65.9	Deep learning model (ECG12Net-based CNN)	14 327 (46.6)	7764 (25.2)	8666 (28.2)	7667 (24.9)
Gibson *et al*.^18^	872	Retrospective observational dataset study	Mexico, Colombia, Brazil	500 (57.3)	NR	56–59	1-D CNN	NR	NR	NR	NR
Herman *et al*.^7^	1032	Retrospective multicenter registry	USA	729 (70.6)	NR	NR	DNN, Queen of Hearts AI ECG model (PMcardio)	NR	NR	NR	NR
ROMIAE Lee *et al*.^6^	8493	Prospective multicentre observational cohort	Republic of Korea	5307 (62.5)	NR	62	AI-ECG model (AiTiAMI v1.00.00)	3923 (46.2)	2117 (24.9)	2313 (27.2)	1762 (20.7)
Zhao *et al*.^11^	8238	Retrospective observational dataset study	China	3366 (40.9)	73.4	NR	DNN (Res-Net architecture)	1545 (18.8)	1305 (15.8)	NR	NR

Sample size represents the number of patients included in each study (patient-level analysis).

AI, artificial intelligence; CAD, coronary artery disease; CNN, convolutional neural network; DL, deep learning; DNN, deep neural network; ECG, electrocardiogram; HTN, hypertension; NR, not reported; USA, United States of America.

### Diagnostic performance of AI in AMI

**Figure 2:**
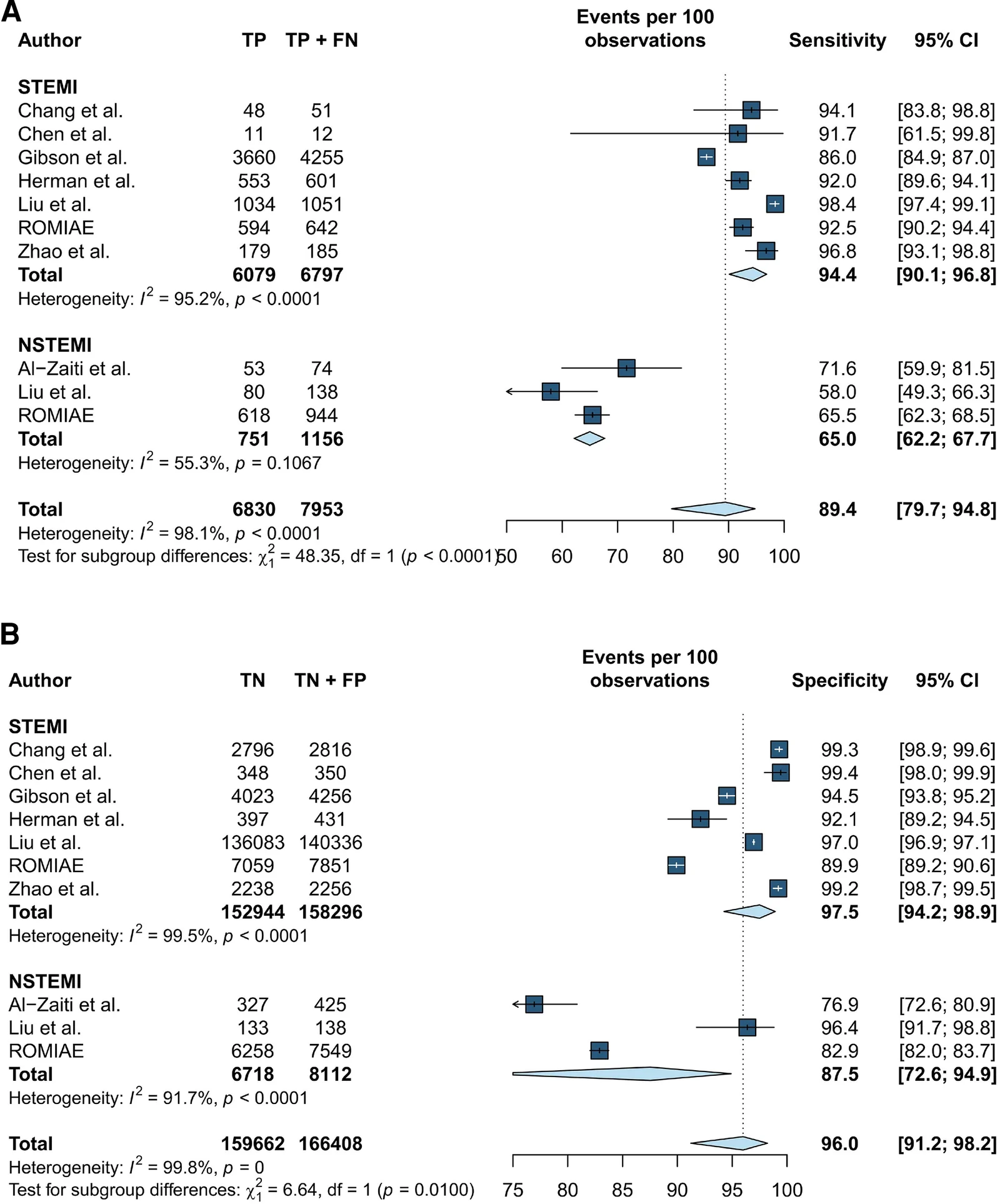
Forest plot of pooled sensitivity and specificity. Diagnostic accuracy estimates were calculated at the patient level using reconstructed 2 × 2 contingency tables. CI, confidence interval; FN, false negative; FP, false positive; TN, true negative; TP, true positive.

**Figure 3:**
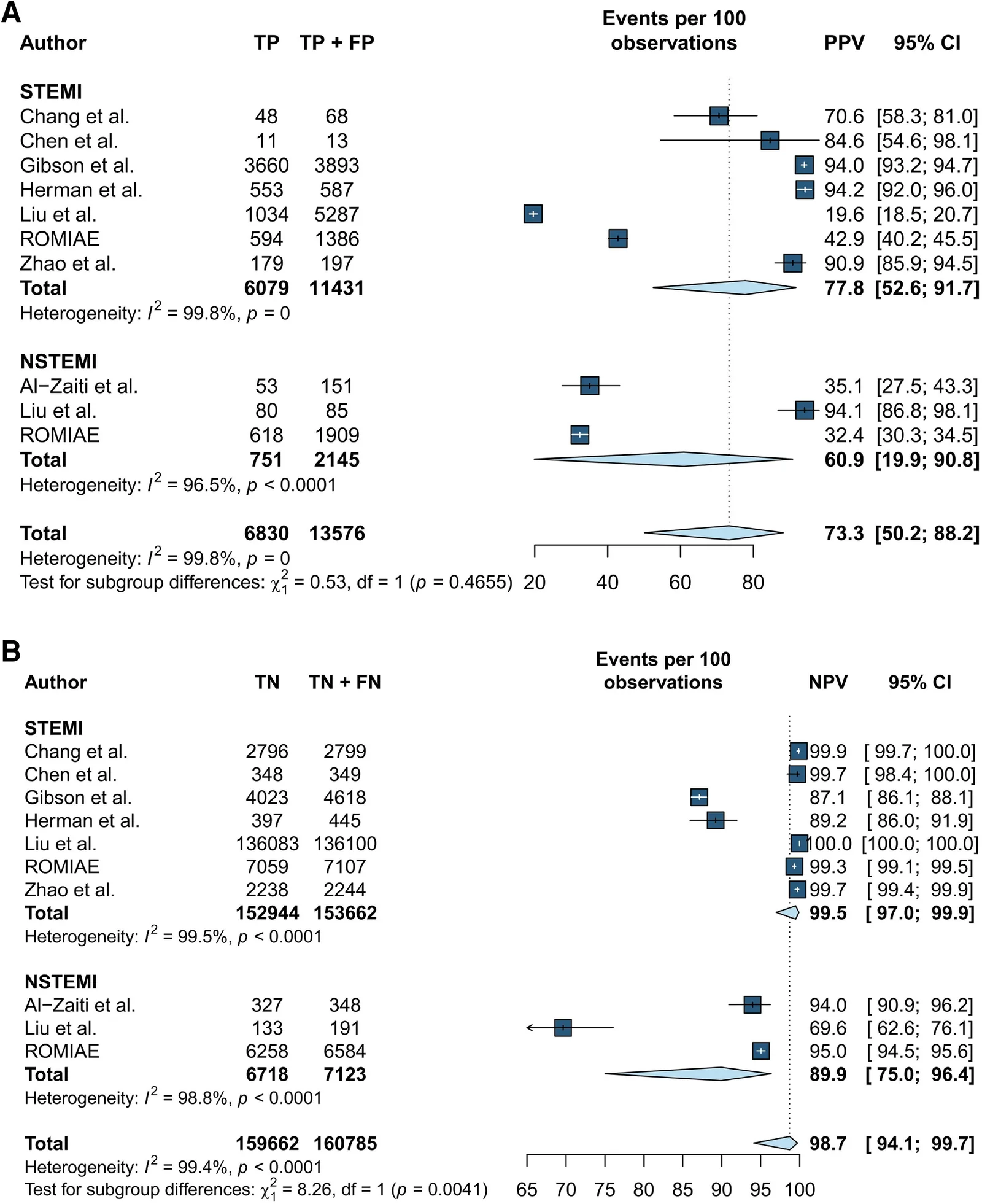
Forest plot of pooled positive predictive value (PPV) and NPV. Diagnostic estimates were calculated at the patient level using reconstructed 2 × 2 contingency tables. CI, confidence interval; FN, false negative; FP, false positive; TN, true negative; TP, true positive.

**Figure 4:**
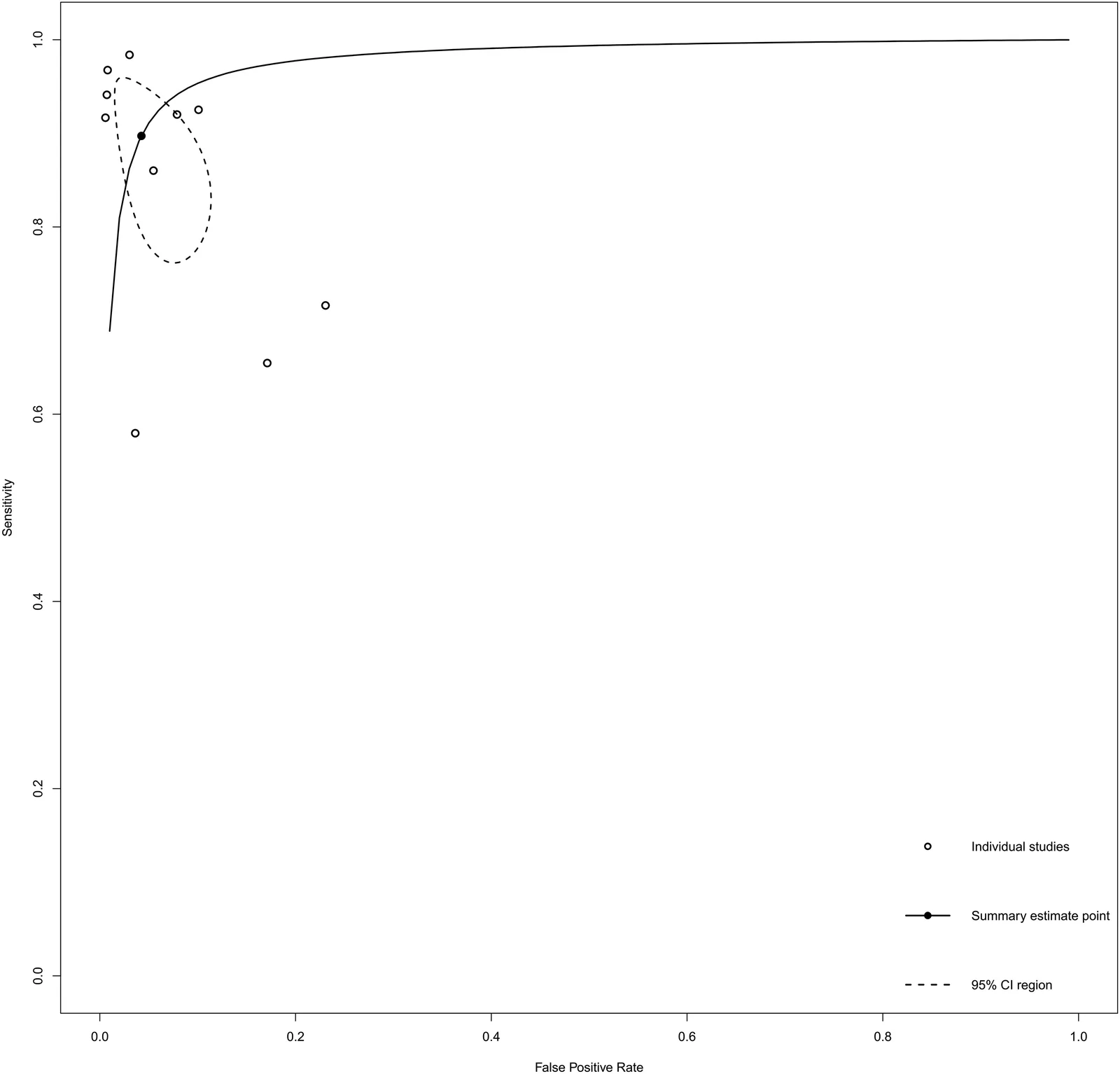
Pooled Summary Receiver-Operating Characteristic (SROC) analysis. CI, confidence interval.

Overall, AI demonstrated a pooled sensitivity of 89.4% (95% CI, 79.7–94.8%; *I*^2^ = 98.1%; *Figure 2A*) and a pooled specificity of 96% (95% CI, 91.2–98.2%; *I*^2^ = 99.8%; *Figure 2B*) for the detection of AMI. The pooled NPV was 98.7% (95% CI, 94.1–99.7%; *I*^2^ = 99.4%; *Figure 3A*), whereas the pooled PPV was 73.3% (95% CI, 50.2–88.2%; *I*^2^ = 99.8%; *Figure 3B*). SROC analysis demonstrated an AUC of 0.97 (95% CI, 0.92–0.98; *Figure 4*).

### Subgroup analysis of diagnostic performance by AMI subtype

**Figure 5:**
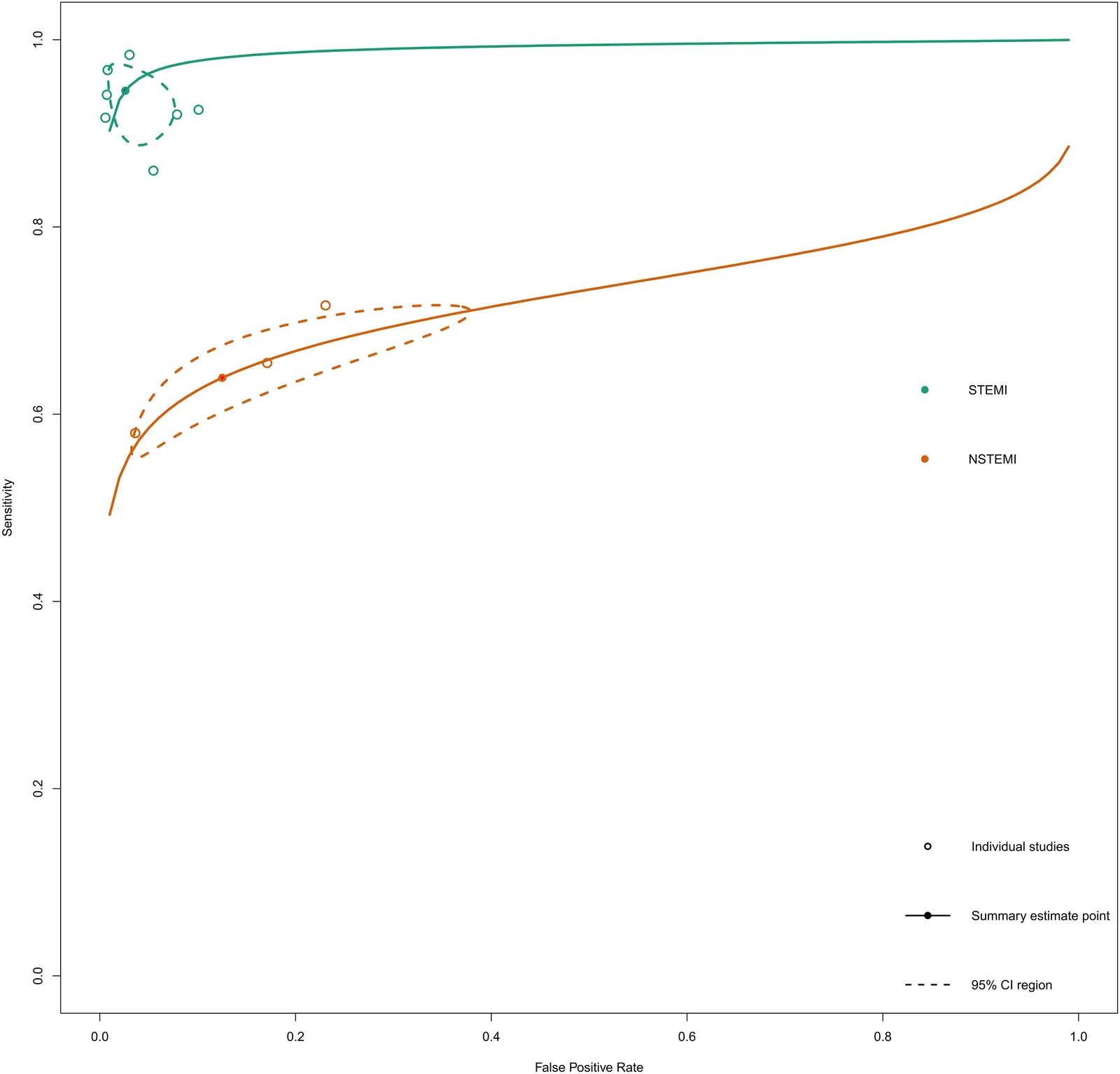
Pooled summary receiver-operating characteristic (SROC) analysis by subgroups. CI, confidence interval; NSTEMI, non-ST-segment elevation myocardial infarction; STEMI, ST-segment elevation myocardial infarction.

In the subgroup analysis for STEMI, AI demonstrated a pooled sensitivity of 94.4% (95% CI 90.1–96.8; *I*^2^ = 95.2%; *Figure 2A*) and a pooled specificity of 97.5% (95% CI, 94.2–98.9%; *I*^2^ = 99.5%; *Figure 2B*). The pooled NPV was 99.5% (95% CI, 97–99.9%; *I*^2^ = 99.5%; *Figure 3A*), whereas the pooled PPV was 77.8% (95% CI, 52.6–91.7%; *I*^2^ = 99.8%; *Figure 3B*). SROC analysis yielded an AUC of 0.98 (95% CI, 0.94–0.98; *Figure 5*).

For NSTEMI, AI demonstrated a pooled sensitivity of 65.0% (95% CI, 62.2–67.7; *I*^2^ = 55.3%; *Figure 2A*) and a pooled specificity of 87.5% (95% CI, 72.6–94.9%; *I*^2^ = 91.7%; *Figure 2B*). The pooled NPV was 89.9% (95% CI, 75–96.4%; *I*^2^ = 98.8%; *Figure 3A*), whereas the pooled PPV was 60.9% (95% CI, 19.9–90.8; *I*^2^ = 96.5%; *Figure 3B*). SROC analysis yielded an AUC of 0.71 (95% CI, 0.63–0.85; Supplementary material online, *Figure S5*).

### Subgroup analysis of diagnostic performance by study design

In the subgroup analysis of retrospective studies, AI demonstrated a pooled sensitivity of 92.3% (95% CI 83.5–96.6; *I*^2^ = 97%; Supplementary material online, *Figure S6*) and a pooled specificity of 96.9% (95% CI, 93.6–98.5%; *I*^2^ = 99%; Supplementary material online, *Figure S5*). The pooled NPV was 98.9% (95% CI, 92.1–99.9%; *I*^2^ = 100%; Supplementary material online, *Figure S8*), whereas the pooled PPV was 80.2% (95% CI, 55.2–93%; *I*^2^ = 100%; Supplementary material online, *Figure S7*). SROC analysis yielded an AUC of 0.98 (95% CI, 0.94–0.99; Supplementary material online, *Figure S9*).

In prospective studies, AI demonstrated a pooled sensitivity of 66.2% (95% CI, 63.3–69; *I*^2^ = 49%; Supplementary material online, *Figure S6*) and a pooled specificity of 93% (95% CI, 64.1–99%; *I*^2^ = 94%; Supplementary material online, *Figure S5*). The pooled NPV was 97.5% (95% CI, 90.1–99.4%; *I*^2^ = 78%; Supplementary material online, *Figure S8*), whereas the pooled PPV was 47.7% (95% CI, 22.9–73.7; *I*^2^ = 81%; Supplementary material online, *Figure S7*). SROC analysis yielded an AUC of 0.87 (95% CI, 0.56–0.97; Supplementary material online, *Figure S9*).

### Sensitivity analysis

A leave-one-out sensitivity analysis was performed for studies evaluating the STEMI and NSTEMI subgroups (see Supplementary material online, *Figures S1* and *S2*). In the STEMI subgroup, exclusion of individual studies yielded results consistent with the primary analysis, with no substantive changes in the pooled estimates. In contrast, in the NSTEMI subgroup, exclusion of the study by Liu *et al*.^17^ resulted in a marked reduction in heterogeneity for pooled sensitivity, positive predictive value, and NPV, while pooled specificity remained consistent with the primary findings.

### Risk of bias assessment and publication bias assessment

Among ten included studies, five were considered to have a low-risk of bias and another five were considered to have a high overall risk of bias, mainly due to limitations in ‘Domain 1,’ ‘Domain 2,’ and ‘Domain 3’. The high risk in ‘Domain 1’ was mainly associated with inappropriate exclusions leading to selection bias and, consequently, overestimation of diagnostic performance. In addition, the presence of biases in the ‘Index Test’ and ‘Reference Standard’ were a result of the lack of detail on pre-specified thresholds and, especially in the study by Chang *et al*.,^14^ due to a misclassification bias due to the lack of angiographic confirmation for all cases of acute ST-segment elevation myocardial infarction presented by the study population.

Regarding applicability, the studies by Choi *et al*.^12^; Chang *et al*.^14^; Chen *et al*.^15^; Zhao *et al*.^11^ and Liu *et al*.^17^ were assessed as having a high risk of bias, mainly due to patient selection, as they were mostly conducted in single, highly complex centres, which confers high geographic specificity, making it difficult to generalize the results achieved to other clinical settings. The risk of bias is illustrated in Supplementary material online, *Figure S3*. Publication bias was evaluated using a Deeks’ funnel plot (see Supplementary material online, *Figure S4*), and the Deeks’ funnel plot asymmetry test indicated no evidence of publication bias (*P* = 0.432).

## Discussion

In this diagnostic systematic review and meta-analysis, AI-enabled ECG demonstrated high sensitivity and consistently high NPV for the detection of AMI, with robust performance in STEMI and more modest accuracy in NSTEMI presentations. While several individual studies have reported promising results, our pooled analysis provides a more comprehensive estimate of diagnostic performance across heterogeneous populations and AI model architectures.

The included studies also employed diverse AI architectures, including convolutional neural networks (CNNs), hybrid CNN–long short-term memory (LSTM) models, and proprietary deep learning algorithms. Differences in model architecture, training datasets, preprocessing strategies, and feature extraction approaches may influence diagnostic performance and contribute to the between-study heterogeneity observed in this meta-analysis. In particular, variations in training data composition, model optimization strategies, and external validation procedures may affect how algorithms generalize across different patient populations and clinical environments. These factors should be considered when interpreting pooled estimates of diagnostic accuracy and highlight the importance of prospective external validation of AI-ECG models in diverse clinical settings.

Importantly, the meta-analytic approach also allowed direct comparison between AMI subtypes, highlighting the substantially higher accuracy observed in STEMI compared with NSTEMI. These results suggest that AI-ECG may serve as a rapid, non-invasive triage tool to support early rule-out of AMI and assist in identifying patients who require expedited evaluation for reperfusion therapy. Given the well-established association between ischaemic time, infarct size, survival, and subsequent heart failure risk, earlier diagnostic clarification may have important clinical implications.

The lower diagnostic performance observed for NSTEMI compared with STEMI likely reflects several pathophysiological and methodological factors. Unlike STEMI, which is typically associated with clear ST-segment elevation patterns on ECG, NSTEMI often presents with subtle or non-specific electrocardiographic abnormalities.^19^ In many cases, myocardial ischaemia is related to non-occlusive coronary events or smaller infarct territories, which may produce minimal or transient electrical changes that are difficult to detect using ECG alone.^20^ In addition, some AI models may be trained on datasets enriched with classic STEMI presentations, potentially biasing model performance toward more overt ischaemic patterns. These factors highlight the intrinsic limitations of ECG-based detection of non-ST-elevation myocardial infarction and underscore the importance of integrating AI-ECG outputs with clinical assessment and cardiac biomarkers.

AI-ECG models typically generate continuous risk scores rather than fixed binary classifications, allowing different operating thresholds to be applied depending on the intended clinical objective. This characteristic may facilitate their integration into multi-threshold clinical decision frameworks similar to those used in high-sensitivity troponin algorithms for suspected ACS.^19^ In such an approach, very low probability outputs could support early rule-out of AMI, whereas high probability outputs could facilitate rapid rule-in and prompt activation of cardiology pathways. Intermediate probability ranges could trigger additional diagnostic testing or clinical reassessment. Although most of the studies included in this meta-analysis evaluated AI-ECG performance using single decision thresholds, future prospective studies should explore predefined multi-threshold strategies to better reflect real-world triage pathways.

The clinical value of AI-ECG is particularly pronounced in time-critical settings where early decisions determine downstream management.^7^ In the prehospital environment, AI-assisted ECG interpretation may enable earlier recognition of STEMI, facilitate prehospital thrombolysis when catheterization is not immediately available, and allow advanced activation of the catheterization laboratory to optimize door-to-balloon times.^1,21^ Similarly, in emergency department triage, AI-ECG may reduce delays caused by diagnostic uncertainty, support early rule-out strategies, and improve the prioritization of invasive evaluation. These system-level effects are especially relevant in high-volume or resource-constrained settings, where delays in expert ECG interpretation remain common.^5,22^

Our findings are consistent with prior observational and prospective studies demonstrating that AI-based ECG analysis outperforms traditional rule-based algorithms and approaches expert-level accuracy in detecting acute ischaemia, including high-risk NSTEMI and occlusive MI phenotypes that frequently lack classic ST-elevation patterns.^6,7^ Importantly, conventional ECG interpretation performs less reliably in atypical presentations, women, older adults, and patients with baseline conduction abnormalities, populations in whom missed or delayed AMI diagnosis remains a major contributor to adverse outcomes. By capturing subtle, multidimensional ECG features beyond human visual assessment, AI-ECG offers a mechanistically plausible solution to these diagnostic gaps.^23,24^

Beyond its role as a diagnostic classifier, AI-enabled ECG may also function as a digital biomarker reflecting underlying cardiac physiology.^25^ Deep learning models can identify subtle electrophysiological signatures associated with myocardial ischaemia, structural heart disease, and other pathophysiological states that may not be visually apparent on standard ECG interpretation^25–27^ This capability suggests that AI-ECG could provide additional biologically meaningful information about myocardial injury and electrical remodelling, potentially supporting earlier detection, improved risk stratification, and more personalized clinical decision-making.

Beyond diagnostic accuracy, AI-enabled ECG may be conceptualized as a tool that captures latent electrophysiological signatures of myocardial ischaemia, reflecting complex interactions between conduction dynamics, repolarization abnormalities, and early myocardial injury.^23–25,30^ Unlike traditional rule-based interpretation, which relies on predefined thresholds, deep learning models integrate multidimensional temporal and spatial electrophysiological features, potentially identifying subclinical ischaemic patterns not readily visible to human interpretation^25–27^.

From a clinical perspective, this capability supports a paradigm shift from binary diagnostic classification toward probabilistic risk stratification at first medical contact.^23,28^ In this context, AI-ECG outputs may inform early triage decisions, enabling rapid rule-out in low-risk patients and prioritization of urgent evaluation in high-risk cases.^6,7^ Such integration within structured clinical pathways, alongside biomarkers and established risk scores, may enhance diagnostic efficiency while maintaining clinician oversight and decision accountability.^22,24^

Importantly, AI-ECG should be viewed as a triage and decision-support tool rather than a replacement for clinical judgment, biomarkers, or imaging. Its greatest utility lies in early risk stratification, identifying patients who can be safely ruled out, those who warrant rapid rule-in pathways, and those who require additional evaluation. Future research should focus on prospective implementation studies evaluating clinical outcomes, workflow integration, cost-effectiveness, and equity across diverse healthcare systems.^28^

Positive predictive value (PPV) and NPV are inherently dependent on disease prevalence and may therefore vary across different clinical settings and patient populations. Because the included studies reported different AMI prevalence rates, pooled estimates of PPV and NPV should be interpreted with caution. In contrast, sensitivity and specificity are less dependent on disease prevalence and may provide more generalizable measures of diagnostic performance across heterogeneous populations.^29^

From a clinical implementation perspective, AI-enabled ECG is best positioned as an adjunctive triage tool rather than a standalone diagnostic replacement. By enabling early risk stratification and supporting rapid rule-out and escalation decisions, AI-ECG has the potential to improve diagnostic efficiency and care prioritization—particularly in high-volume emergency settings and resource-constrained environments. By synthesizing evidence across heterogeneous study designs, populations, and AI architectures, the present meta-analysis provides a more comprehensive estimate of diagnostic performance and highlights key differences in model accuracy across AMI subtypes. These findings help contextualize individual reports and identify important priorities for future prospective validation, external validation, and real-world clinical implementation

### Study limitations

Several limitations warrant consideration. First, the included studies were predominantly observational, with heterogeneity in AI architectures, ECG acquisition protocols, reference standards, and AMI definitions, which may contribute to between-study variability. Second, most studies evaluated diagnostic performance in controlled or retrospective settings, limiting inference regarding real-world implementation, workflow impact, and downstream clinical outcomes. Third, few studies reported granular subgroup analyses by sex, age, comorbidities, or ECG confounders such as bundle branch block or ventricular pacing. Fourth, AI models were generally assessed as stand-alone diagnostic tools, whereas their optimal role is likely as integrated decision support combined with clinical assessment and biomarkers. Finally, reporting on calibration, interpretability, and failure modes was sparse, constraining assessment of generalizability and potential bias. In addition, half of the included studies were analyzed as having a high risk of bias by the QUADAS-2 tool,^10^ especially due to selection biases of the participants involved in the study and geographical restrictions, which limits the generalization of their results.

### Future directions

Future research should move beyond diagnostic accuracy and focus on prospective, multicenter implementation studies assessing the impact of AI-ECG–guided triage on ischaemic time, clinical outcomes, and healthcare utilization. Integration of AI-ECG with high-sensitivity troponin assays, clinical risk scores, and prehospital workflows represents a key step toward streamlined, time-efficient AMI care pathways.

In parallel, standardized reporting of calibration, explainability, and subgroup performance will be essential to ensure transparency, generalizability, and equitable deployment across populations and healthcare systems. Health economic analyses are also needed to define the value proposition of AI-ECG in different clinical environments. Ultimately, randomized or pragmatic trials comparing AI-assisted triage with standard care will be required to inform guideline recommendations and determine its role in contemporary AMI management.

## Conclusion

AI-enabled electrocardiography demonstrated consistently high sensitivity and NPV for AMI across STEMI and NSTEMI presentations. These findings support its potential role as a non-invasive triage adjunct to assist early diagnostic decision-making and patient prioritization at first medical contact.

## Supplementary Material

ztag075_Supplementary_Data

## Data Availability

The data used in this meta-analysis were extracted from publicly available sources, including published observational and randomized controlled trials. All relevant data supporting the findings of this study are included within the manuscript and supplementary materials. Due to the nature of this analysis, no new primary data were generated. Additional details can be made available upon reasonable request to the corresponding author.

## References

[1] Rao SV, O’Donoghue ML, Ruel M, Rab T, Tamis-Holland JE, Alexander JH, et al 2025 ACC/AHA/ACEP/NAEMSP/SCAI Guideline for the management of patients with acute coronary syndromes: a report of the American College of Cardiology/American Heart Association Joint Committee on Clinical Practice Guidelines. J Am Coll Cardiol 2025;85:2135–2237. 40013746 10.1016/j.jacc.2024.11.009

[2] Tzimas G, Antiochos P, Monney P, Eeckhout E, Meier D, Fournier S, et al Atypical electrocardiographic presentations in need of primary percutaneous coronary intervention. Am J Cardiol 2019;124:1305–1314. 31455501 10.1016/j.amjcard.2019.07.027

[3] Miranda DF, Lobo AS, Walsh B, Sandoval Y, Smith SW. New insights into the use of the 12-lead electrocardiogram for diagnosing acute myocardial infarction in the emergency department. Can J Cardiol 2018;34:132–145. 29407007 10.1016/j.cjca.2017.11.011

[4] Kim EJ, Gala D, Ayyad M, Pramanik M, Makaryus AN. AI applications in electrocardiography for ischemic and structural heart disease: a review of the current state. J Clin Med 2026;15:316. 41517565 10.3390/jcm15010316 PMC12786525

[5] Kim J, Shon B, Kim S, Cho J, Seo JJ, Jang SY, et al ECG data analysis to determine ST-segment elevation myocardial infarction and infarction territory type: an integrative approach of artificial intelligence and clinical guidelines. Front Physiol 2024;15:1462847. 39434722 10.3389/fphys.2024.1462847 PMC11491539

[6] Lee MS, Shin TG, Lee Y, Kim DH, Choi SH, Cho H, et al Artificial intelligence applied to electrocardiogram to rule out acute myocardial infarction: the ROMIAE multicentre study. Eur Heart J 2025;46:1917–1929. 39992309 10.1093/eurheartj/ehaf004 PMC12093146

[7] Herman R, Mumma BE, Hoyne JD, Cooper BL, Johnson NP, Kisova T, et al AI-Enabled ECG analysis improves diagnostic accuracy and reduces false STEMI activations: a multicenter U.S. Registry. JACC Cardiovasc Interv 2026;19:145–156. 41158088 10.1016/j.jcin.2025.10.018

[8] In: Higgins JPT, Thomas J, Chandler J, Cumpston M, Li T, Page MJ, et al, eds. Cochrane Handbook for Systematic Reviews of Interventions Version 6.5 (Updated August 2024): Cochrane; 2024. www.cochrane.org/handbook.

[9] Page MJ, McKenzie JE, Bossuyt PM, Boutron I, Hoffmann TC, Mulrow CD, et al The PRISMA 2020 statement: an updated guideline for reporting systematic reviews. BMJ 2021;372:n71. 33782057 10.1136/bmj.n71 PMC8005924

[10] Whiting PF, Rutjes AWS, Westwood ME, Mallett S, Deeks JJ, Reitsma JB, et al QUADAS-2: a revised tool for the quality assessment of diagnostic accuracy studies. Ann Intern Med 2011;155:529–536. 22007046 10.7326/0003-4819-155-8-201110180-00009

[11] Zhao Y, Xiong J, Hou Y, Zhu M, Lu Y, Xu Y, et al Early detection of ST-segment elevated myocardial infarction by artificial intelligence with 12-lead electrocardiogram. Int J Cardiol 2020;317:223–230. 32376417 10.1016/j.ijcard.2020.04.089

[12] Choi JWH, Torelli V, Silverman A, Diaz SS, Kong D, Vaish E, et al AI-enhanced recognition of occlusions in acute coronary syndrome (AERO-ACS): a retrospective study. Coron Artery Dis 2026;37:39–45. 40705383 10.1097/MCA.0000000000001555

[13] Al-Zaiti S, Besomi L, Bouzid Z, Faramand Z, Frisch S, Martin-Gill C, et al Machine learning-based prediction of acute coronary syndrome using only the pre-hospital 12-lead electrocardiogram. Nat Commun 2020;11:3966. 32769990 10.1038/s41467-020-17804-2 PMC7414145

[14] Chang KC, Hsieh PH, Wu MY, Wang YC, Wei JT, Shih ESC, et al Usefulness of multi-labelling artificial intelligence in detecting rhythm disorders and acute ST-elevation myocardial infarction on 12-lead electrocardiogram. Eur Heart J Digit Health 2021;2:299–310. 36712388 10.1093/ehjdh/ztab029 PMC9708016

[15] Chen KW, Wang YC, Liu MH, Tsai BY, Wu MY, Hsieh PH, et al Artificial intelligence-assisted remote detection of ST-elevation myocardial infarction using a mini-12-lead electrocardiogram device in prehospital ambulance care. Front Cardiovasc Med 2022;9:1001982. 36312246 10.3389/fcvm.2022.1001982 PMC9614054

[16] Riek NT, Akcakaya M, Bouzid Z, Gokhale T, Helman SM, Kraevsky K, et al ECG-SMART-NET: a deep learning architecture for precise ECG diagnosis of occlusion myocardial infarction. IEEE Trans Biomed Eng 2025;72:3613–3620. 40418608 10.1109/TBME.2025.3573581 PMC12747319

[17] Liu WC, Lin CS, Tsai CS, Tsao TP, Cheng CC, Liou JT, et al A deep learning algorithm for detecting acute myocardial infarction. EuroIntervention 2021;17:765–773. 33840640 10.4244/EIJ-D-20-01155 PMC9724911

[18] Gibson CM, Mehta S, Ceschim MRS, Frauenfelder A, Vieira D, Botelho R, et al Evolution of single-lead ECG for STEMI detection using a deep learning approach. Int J Cardiol 2022;346:47–52. 34801613 10.1016/j.ijcard.2021.11.039

[19] Byrne RA, Rossello X, Coughlan JJ, Barbato E, Berry C, Chieffo A, et al 2023 ESC guidelines for the management of acute coronary syndromes. Eur Heart J 2023;44:3720–3826. 37622654 10.1093/eurheartj/ehad191

[20] Thygesen K, Alpert JS, Jaffe AS, Chaitman BR, Bax JJ, Morrow DA, et al Fourth Universal Definition of Myocardial Infarction (2018). Circulation 2018;138:e618–e651. 30571511 10.1161/CIR.0000000000000617

[21] Faour A, Cherrett C, Gibbs O, Lintern K, Mussap CJ, Rajaratnam R, et al Utility of prehospital electrocardiogram interpretation in ST-segment elevation myocardial infarction utilizing computer interpretation and transmission for interventional cardiologist consultation. Catheter Cardiovasc Interv 2022;100:295–303. 35766040 10.1002/ccd.30300 PMC9546148

[22] Wang YC, Chen KW, Tsai BY, Wu MY, Hsieh PH, Wei JT, et al Implementation of an all-day artificial intelligence-based triage system to accelerate door-to-balloon times. Mayo Clin Proc 2022;97:2291–2303. 36336511 10.1016/j.mayocp.2022.05.014

[23] Lin CS, Liu WT, Chen YH, Lin SH, Lin C. Artificial intelligence-enabled electrocardiography from scientific research to clinical application. EMBO Mol Med 2026;18:22–40. 41326714 10.1038/s44321-025-00351-y PMC12808761

[24] Armoundas AA, Narayan SM, Arnett DK, Spector-Bagdady K, Bennett DA, Celi LA, et al Use of artificial intelligence in improving outcomes in heart disease: a scientific statement from the American Heart Association. Circulation 2024;149:e1028–e1050. 38415358 10.1161/CIR.0000000000001201 PMC11042786

[25] Attia ZI, Kapa S, Noseworthy PA, Lopez-Jimenez F, Friedman PA. Artificial intelligence ECG to detect left ventricular dysfunction in COVID-19: a case series. Mayo Clin Proc 2020;95:2464–2466. 33153634 10.1016/j.mayocp.2020.09.020 PMC7501873

[26] Kim KH, Kwon JM, Pereira T, Attia ZI, Pereira NL. Artificial intelligence applied to cardiomyopathies: is it time for clinical application? Curr Cardiol Rep 2022;24:1547–1555. 36048306 10.1007/s11886-022-01776-4

[27] Siontis KC, Noseworthy PA, Attia ZI, Friedman PA. Artificial intelligence-enhanced electrocardiography in cardiovascular disease management. Nat Rev Cardiol 2021;18:465–478. 33526938 10.1038/s41569-020-00503-2 PMC7848866

[28] Bdir S, Jaber M, Tanbouz O, Milhem F, Sarhan I, Bdair M, et al Artificial intelligence for myocardial infarction detection via electrocardiogram: a scoping review. J Clin Med 2025;14:6792. 41095870 10.3390/jcm14196792 PMC12525322

[29] Bossuyt PM, Reitsma JB, Bruns DE, Gatsonis CA, Glasziou PP, Irwig L, et al STARD 2015: an updated list of essential items for reporting diagnostic accuracy studies. Clin Chem 2015;61:1446–1452. 26510957 10.1373/clinchem.2015.246280

[30] Kwon JM, Jung MS, Kim KH, Jo YY, Shin JH, Cho YH, et al Artificial intelligence for detecting electrolyte imbalance using electrocardiography. Ann Noninvasive Electrocardiol 2021;26:e12839. 33719135 10.1111/anec.12839 PMC8164149

