# Supplementary material for: Artificial intelligence–based ECG as a triage tool for acute myocardial infarction: a diagnostic systematic review and meta-analysis
Source: Eur Heart J Digit Health. 2026 Aug 29;7(7):ztag075. doi: 10.1093/ehjdh/ztag075 (PMC13525340; doi:10.1093/ehjdh/ztag075)
Supplement: Supplementary file 1 [file ztag075_supplementary_data.docx]

Supplementary Materials

**Supplemental Methods 1. PRISMA 2020 Main Checklist[1]	2**

**Supplemental Methods 2. PRISMA Abstract Checklist[1]	3**

**Supplemental Methods 3. Details of the Search Strategy	4**

**Supplemental Table 1. Eligibility Criteria of the included studies	6**

**Supplemental Figure 1. Forest Plot leave-one-out sensitivity analysis for Non-STEMI	9**

**Supplemental Figure 2. Forest Plot leave-one-out sensitivity analysis for STEMI	11**

**Supplemental Figure 3. Risk of Bias Assessment with QUADAS-2 tool	14**

**Supplemental Figure 4. Deeks’ Funnel Plot for Publication Bias	15**

**Supplemental Figure 5. Forest plot of specificity according to study design.	16**

**Supplemental Figure 6. Forest plot of pooled sensitivity according to study design.	17**

**Supplemental Figure 7. Forest plot of pooled positive predictive value (PPV) according to study design.	18**

**Supplemental Figure 8. Forest plot of pooled negative predictive value (NPV) according to study design.	19**

**Supplemental Figure 9. Summary receiver operating characteristic (SROC) curves according to study design.	20**

**References	22**

# Supplemental Methods 1. PRISMA 2020 Main Checklist[1]

| **Topic** | **No.** | **Item** | **Location where item is reported** |
| --- | --- | --- | --- |
| **TITLE** |  |  |  |
| **Title** | 1 | Identify the report as a systematic review. | Pg.1, MS |
| **ABSTRACT** |  |  |  |
| **Abstract** | 2 | See the PRISMA 2020 for Abstracts checklist | Pg.1, MS |
| **INTRODUCTION** |  |  |  |
| **Rationale** | 3 | Describe the rationale for the review in the context of existing knowledge. | Pg.3, MS |
| **Objectives** | 4 | Provide an explicit statement of the objective(s) or question(s) the review addresses. | Pg.3, MS |
| **METHODS** |  |  |  |
| **Eligibility criteria** | 5 | Specify the inclusion and exclusion criteria for the review and how studies were grouped for the syntheses. | Pg.4, MS |
| **Information sources** | 6 | Specify all databases, registers, websites, organizations, reference lists and other sources searched or consulted to identify studies. Specify the date when each source was last searched or consulted. | Pg. 4, MS and Supplementary Methods 6 |
| **Search strategy** | 7 | Present the full search strategies for all databases, registers, and websites, including any filters and limits used. | Pg. 4, MS and Supplementary Methods 6 |
| **Selection process** | 8 | Specify the methods used to decide whether a study met the inclusion criteria of the review, including how many reviewers screened each record and each report retrieved, whether they worked independently, and if applicable, details of automation tools used in the process. | Pg.4, MS |
| **Data collection process** | 9 | Specify the methods used to collect data from reports, including how many reviewers collected data from each report, whether they worked independently, any processes for obtaining or confirming data from study investigators, and if applicable, details of automation tools used in the process. | Pg.4, MS |
| **Data items** | 10a | List and define all outcomes for which data were sought. Specify whether all results that were compatible with each outcome domain in each study were sought (e.g., for all measures, time points, analyses), and if not, the methods used to decide which results to collect. | Pg.4, MS |
|  | 10b | List and define all other variables for which data were sought (e.g., participant and intervention characteristics, funding sources). Describe any assumptions made about any missing or unclear information. | Pg.4, MS |
| **Study risk of bias assessment** | 11 | Specify the methods used to assess risk of bias in the included studies, including details of the tool(s) used, how many reviewers assessed each study and whether they worked independently, and if applicable, details of automation tools used in the process. | Pg.4, MS |
| **Effect measures** | 12 | Specify for each outcome the effect measure(s) (e.g., risk ratio, mean difference) used in the synthesis or presentation of results. | Pg.4, MS |
| **Synthesis methods** | 13a | Describe the processes used to decide which studies were eligible for each synthesis (e.g., tabulating the study intervention characteristics and comparing against the planned groups for each synthesis (item 5)). | Pg.4, MS |
|  | 13b | Describe any methods required to prepare the data for presentation or synthesis, such as handling of missing summary statistics, or data conversions. | Pg.4, MS |
|  | 13c | Describe any methods used to tabulate or visually display results of individual studies and syntheses. | Pg.4, MS |
|  | 13d | Describe any methods used to synthesize results and provide a rationale for the choice(s). If meta-analysis was performed, describe the model(s), method(s) to identify the presence and extent of statistical heterogeneity, and software package(s) used. | Pg. 4 MS |
|  | 13e | Describe any methods used to explore possible causes of heterogeneity among study results (e.g., subgroup analysis, meta-regression). | Pg. 4, MS and Pg. 9-12, Supplementary |
|  | 13f | Describe any sensitivity analyses conducted to assess robustness of the synthesized results. | Pg. 4, MS and Pg. 9-12, Supplementary |
| **Reporting bias assessment** | 14 | Describe any methods used to assess risk of bias due to missing results in a synthesis (arising from reporting biases). | Pg. 4-5, MS |
| **Certainty assessment** | 15 | Describe any methods used to assess certainty (or confidence) in the body of evidence for an outcome. | NA |
| **RESULTS** |  |  |  |
| **Study selection** | 16a | Describe the results of the search and selection process, from the number of records identified in the search to the number of studies included in the review, ideally using a flow diagram. | Pg.5, MS, Figure 1 |
|  | 16b | Cite studies that might appear to meet the inclusion criteria, but which were excluded, and explain why they were excluded. | NA |
| **Study characteristics** | 17 | Cite each included study and present its characteristics. | Table 1, Pg.5, MS |
| **Risk of bias in studies** | 18 | Present assessments of risk of bias for each included study. | Pg. 6, MS and Pg. 13, Supplementary |
| **Results of individual studies** | 19 | For all outcomes, present, for each study: (a) summary statistics for each group (where appropriate) and (b) an effect estimates and its precision (e.g., confidence/credible interval), ideally using structured tables or plots. | Fig. 2-4; Pg. 5, MS and Pg. 9-12, Supplementary |
| **Results of syntheses** | 20a | For each synthesis, briefly summarize the characteristics and risk of bias among contributing studies. | Pg. 13, Supplementary |
|  | 20b | Present results of all statistical syntheses conducted. If meta-analysis was done, present for each the summary estimate and its precision (e.g., confidence/credible interval) and measures of statistical heterogeneity. If comparing groups, describe the direction of the effect. | Pg 5, MS |
|  | 20c | Present results of all investigations of possible causes of heterogeneity among study results. | Pg. 5, MS and Pg. 9-12, Supplementary |
|  | 20d | Present results of all sensitivity analyses conducted to assess the robustness of the synthesized results. | Pg. 5-6, MS and Pg. 9-12 Supplementary |
| **Reporting biases** | 21 | Present assessments of risk of bias due to missing results (arising from reporting biases) for each synthesis assessed. | Pg 6, MS and Pg. 13 Supplementary |
| **Certainty of evidence** | 22 | Present assessments of certainty (or confidence) in the body of evidence for each outcome assessed. | NA |
| **DISCUSSION** |  |  |  |
| **Discussion** | 23a | Provide a general interpretation of the results in the context of other evidence. | Pg. 6-7, MS |
|  | 23b | Discuss any limitations of the evidence included in the review. | Pg. 7, MS |
|  | 23c | Discuss any limitations of the review processes used. | Pg. 7, MS |
|  | 23d | Discuss implications of the results for practice, policy, and future research. | Pg. 7, MS |
| **OTHER INFORMATION** |  |  |  |
| **Registration and protocol** | 24a | Provide registration information for the review, including register name and registration number, or state that the review was not registered. | PROSPERO; **CRD420261292271** |
|  | 24b | Indicate where the review protocol can be accessed, or state that a protocol was not prepared. | https://www.crd.york.ac.uk/PROSPERO/view/CRD420261292271 |
|  | 24c | Describe and explain any amendments to information provided at registration or in the protocol. | NA |
| **Support** | 25 | Describe sources of financial or non-financial support for the review, and the role of the funders or sponsors in the review. | None |
| **Competing interests** | 26 | Declare any competing interests of review authors. | Pg. 7, MS |
| **Availability of data, code and other materials** | 27 | Report which of the following are publicly available and where they can be found template data collection forms; data extracted from included studies; data used for all analyses; analytic code; any other materials used in the review. | NA |

^Abbreviations: MS, manuscript; sup., supplement.^

# Supplemental Methods 2. PRISMA Abstract Checklist[1]

| **Topic** | **No.** | **Item** | **Reported?** |
| --- | --- | --- | --- |
| **TITLE** |  |  |  |
| **Title** | 1 | Identify the report as a systematic review. | Yes |
| **BACKGROUND** |  |  |  |
| **Objectives** | 2 | Provide an explicit statement of the main objective(s) or question(s) the review addresses. | Yes |
| **METHODS** |  |  |  |
| **Eligibility criteria** | 3 | Specify the inclusion and exclusion criteria for the review. | Yes |
| **Information sources** | 4 | Specify the information sources (e.g., databases, registers) used to identify studies and the date when each was last searched. | Yes |
| **Risk of bias** | 5 | Specify the methods used to assess risk of bias in the included studies. | No |
| **Synthesis of results** | 6 | Specify the methods used to present and synthesize results. | Yes |
| **RESULTS** |  |  |  |
| **Included studies** | 7 | Give the total number of included studies and participants and summarize relevant characteristics of studies. | Yes |
| **Synthesis of results** | 8 | Present results for main outcomes, preferably indicating the number of included studies and participants for each. If meta-analysis was done, report the summary estimate and confidence/credible interval. If comparing groups, indicate the direction of the effect (i.e., which group is favored). | Yes |
| **DISCUSSION** |  |  |  |
| **Limitations of evidence** | 9 | Provide a brief summary of the limitations of the evidence included in the review (e.g., study risk of bias, inconsistency and imprecision). | No |
| **Interpretation** | 10 | Provide a general interpretation of the results and important implications. | Yes |
| **OTHER** |  |  |  |
| **Funding** | 11 | Specify the primary source of funding for the review. | No |
| **Registration** | 12 | Provide the register name and registration number. | Yes |

# Supplemental Methods 3. Details of the Search Strategy

| **Search Strategy for each database** | |
| --- | --- |
| **Pubmed** | (("Myocardial Infarction" OR "Coronary Occlusion" OR  "Acute Coronary Syndrome" OR AMI OR  "acute coronary syndrome*" OR ACS OR "coronary occlusion*" OR  "occlusion MI" OR OMI OR STEMI OR "ST-segment elevation"))  AND  (("Electrocardiography" OR electrocardiograph OR ECG OR EKG))  AND  (("Artificial Intelligence" OR "Machine Learning" OR "Deep Learning" OR  "Neural Networks, Computer"[Mesh] OR "Algorithms" OR  "artificial intelligence" OR "machine learning" OR "deep learning" OR  algorithm* OR "neural network*" OR AI OR "AI-based"))  AND  (("Sensitivity and Specificity" OR "ROC Curve" OR  sensitivity OR specificity OR "area under the curve" OR AUC OR  accuracy OR "diagnostic performance" OR "predictive value*" OR  "likelihood ratio*")) |
| **EMBASE** | (('Myocardial Infarction' OR 'Coronary Occlusion' OR  'Acute Coronary Syndrome' OR AMI OR  'acute coronary syndrome*' OR ACS OR 'coronary occlusion*' OR  'occlusion MI' OR OMI OR STEMI OR 'ST-segment elevation'))  AND  ((Electrocardiography OR electrocardiograph OR ECG OR EKG))  AND  (('Artificial Intelligence' OR 'Machine Learning' OR 'Deep Learning' OR  'Neural Networks, Computer'/exp OR Algorithms OR  'artificial intelligence' OR 'machine learning' OR 'deep learning' OR  algorithm* OR 'neural network*' OR AI OR AI-based))  AND  (('Sensitivity and Specificity' OR 'ROC Curve' OR  sensitivity OR specificity OR 'area under the curve' OR AUC OR  accuracy OR 'diagnostic performance' OR 'predictive value*' OR  'likelihood ratio*')) |
| **Cochrane Library** | (("Myocardial Infarction" OR "Coronary Occlusion" OR  "Acute Coronary Syndrome" OR AMI OR  "acute coronary syndrome*" OR ACS OR "coronary occlusion*" OR  "occlusion MI" OR OMI OR STEMI OR "ST-segment elevation"))  AND  (("Electrocardiography" OR electrocardiograph OR ECG OR EKG))  AND  (("Artificial Intelligence" OR "Machine Learning" OR "Deep Learning" OR  "Neural Networks, Computer"[Mesh] OR "Algorithms" OR  "artificial intelligence" OR "machine learning" OR "deep learning" OR  algorithm* OR "neural network*" OR AI OR "AI-based"))  AND  (("Sensitivity and Specificity" OR "ROC Curve" OR  sensitivity OR specificity OR "area under the curve" OR AUC OR  accuracy OR "diagnostic performance" OR "predictive value*" OR  "likelihood ratio*")) |

# Supplemental Table 1. Eligibility Criteria of the included studies

**ELIGIBILITY CRITERIA TABLE**

| **Study** | **Exclusion Criteria** | **Inclusion Criteria** |
| --- | --- | --- |
| **Choi et al. (AERO-ACS)[2]** | - Lack of a pre-CAG ECG in the system - undetermined to have   ACS from the emergency department   - Transfer from an outside hospital - No documented peak troponin - Diagnosis of unstable angina after negative serial troponins | - Initial presentation suspected to be ACS in the emergency department - Precatheterization ECGs available for AI analysis - No transfer from an outside hospital to ensure consistent access to data. |
| **Al-Zaiti 2020[3]** | - Excessive noise or artifact - ECGs recorded during ventricular tachycardia or ventricular fibrillation | - Consecutive, non-traumatic patients with chest pain transported by emergency medical services - Prehospital 10-second, 12-lead ECG obtained at first medical contact - ECG transmitted to the medical command center and stored as raw digital data - Patients enrolled from three tertiary-care hospitals within the EMPIRE prospective cohort study - Interpretable ECGs, including those with: Bundle branch block, Ventricular pacing, Atrial fibrillation, Left ventricular hypertrophy - Availability of 30-day clinical follow-up data - All ECGs included regardless of ST-segment elevation status (STEMI and NSTE-ACS both included) |
| **Chang et al.[4]** | - Duplicate ECG records - ECGs with incomplete age information or patients <18 years old - ECGs without a definite cardiologist-confirmed diagnosis - ECGs not performed at China Medical University Hospital | - ECGs stored in the digital ECG database of China Medical University Hospital, Taiwan - ECGs recorded between January 15, 2009 and December 31, 2018 - ECGs labelled by board-certified cardiologists - ECGs corresponding to: Acute STEMI, and/or Normal sinus rhythm, and/or 12 rhythm or conduction disorders (e.g., AF, AFL, AV blocks, PSVT, ventricular ectopy) - Adult patients (≥18 years) |
| **Chen et al.[5]** | - Traumatic chest or abdominal injury - Conscious disturbance, failure to cooperate with or refusal of ECG examinations - age ≤18-year-old - Pregnant women | - Patients who called 119 dispatch centers of fire stations for symptoms of chest pain or shortness of breath in Taichung City and Nantou County with performed on-site 12-lead ECG examinations using mini portable devices (QT Medical, Diamond Bar, CA., USA), after pre-on-board training by Emergency medical technicians from the 14 pre-assigned fire stations. |
| **Riek et al. (ECG-Smart-NET)[6]** | - ECGs from patients < 18 years old - ECGs with ventricular tachycardia - ECGs with missing or noisy leads - Serial ECGs removed from validation and test sets | - Adults ≥ 18 years old - Patients with a primary complaint of non-traumatic chest pain - Prehospital 12-lead ECGs collected by EMS - ECGs recorded by three EMS systems:   Pittsburgh EMS (PA), Orange County EMS (NC), Mecklenburg County EMS (NC) |
| **Liu et al.[7]** | - AMI patients without electronic ECG records - ECGs recorded with: Right-sided ECG leads, Posterior ECG leads, Pacemaker rhythm - Non-AMI patients with: History of prior myocardial infarction, or Any elevation of cardiac troponin I during the ED stay | - AMI patients presenting to the emergency department who received a coronary angiogram to rule in type I AMI and to confirm the infarct-related artery of STEMI |
| **Gibson et al.[8]** | - Baseline artifacts | - Standard, 12‐lead ECG tracings obtained from Acute Coronary Syndrome patients for whom ITMS provided telemedicine services during a four-year period between 2014 and 2018. - ECGs classified as: STEMI , or Not-STEMI, including both: Normal ECGs, Abnormal ECGs (non-STEMI abnormalities) |
| **Herman et al.[9]** | - Declined or deferred activation; - Non–acute coronary syndrome CCL activation; - Insufficient ECG quality; - Ventricular arrhythmia on index electrocardiography. | - Emergent CCL activation during index hospitalization - Availability of the index 12-lead electrocardiogram; - Age ≥18 years. |
| **Lee et al. (ROMIAE)[10]** | - Patients presenting with out-of-hospital cardiac arrest upon ED arrival - Traumatic chest pain - Conditions clearly distinct from myocardial infarction (MI) (such as pneumothorax). - Refusal of consent to enroll in the study or unable to be asked about consent | - Adult patients (>18 years old) - Presentation to the emergency department (ED) with clinically suspected acute myocardial infarction (AMI) - Arrival to the ED within 24 hours of: Initial chest pain onset, or worsening chest pain within the prior 24 hours, or recurrent symptoms within 24 hours after ED admission |
| **Zhao et al.[11]** | - No significant ST-segment elevation evaluated by 3 experts - CAG not performed - No definite culprit vessel on CAG - Poor image quality or no definite diagnosis in control ECGs | - STEMI ECGs validated by coronary angiography (CAG) - Raw 12-lead ECG data - Data from inpatients at two Shanghai hospitals (2008–2018) - Control ECGs including normal and abnormal ECGs (non-STEMI conditions) |

- **Abbreviations:** ACS: acute coronary syndrome; AF: atrial fibrillation; AFL: atrial flutter; AI: artificial intelligence; AV blocks: atrioventricular blocks; CAG: coronary angiography; CCL: cardiac catheterization laboratory; ECG: electrocardiogram; ED: emergency department; EMS: emergency medical services; ITMS: International Telemedicine Systems; Non-AMI: non–acute myocardial infarction; NSTE-ACS: non–ST-segment elevation acute coronary syndrome; NSTEMI (Non-STEMI): non–ST-segment elevation myocardial infarction; PSVT: paroxysmal supraventricular tachycardia; STEMI: ST-segment elevation myocardial infarction.

# Supplemental Figure 1. Forest Plot leave-one-out sensitivity analysis for Non-STEMI

A)

B)

C)

D)

Leave-one-out sensitivity analyses for diagnostic performance measures in non-STEMI, including (A) negative predictive value (NPV), (B) positive predictive value (PPV), (C) sensitivity, and (D) specificity. For each outcome, pooled estimates and corresponding 95% confidence intervals were recalculated after sequential exclusion of individual studies using random-effects models.

Abbreviations: Non-STEMI: Non-ST segment elevation Myocardial Infarction CI: confidence interval; NPV: Negative Predictive Value; PPV: Positive Predictive Value

# Supplemental Figure 2. Forest Plot leave-one-out sensitivity analysis for STEMI

**A)**

**B)**

**C)**

**D)**

Leave-one-out sensitivity analyses for diagnostic performance measures in STEMI, including (A) negative predictive value (NPV), (B) positive predictive value (PPV), (C) sensitivity, and (D) specificity. For each outcome, pooled estimates and corresponding 95% confidence intervals were recalculated after sequential exclusion of individual studies using random-effects models.

Abbreviations: STEMI: ST – segment elevation Myocardial Infarction CI: confidence interval; NPV: Negative Predictive Value; PPV: Positive Predictive Value

# Supplemental Figure 3. Risk of Bias Assessment with QUADAS-2 tool

**A)**

**B)**

(A) Risk of bias assessment with QUADAS-2 tool; (B) Applicability concerns with QUADAS-2 tool.

# Supplemental Figure 4. Deeks’ Funnel Plot for Publication Bias

**Supplemental Figure 4. Deeks’ funnel plot for publication bias assessment.**

Deeks’ funnel plot assessing potential publication bias across the included studies in this diagnostic meta-analysis. The Deeks’ asymmetry test did not demonstrate significant evidence of publication bias (p = 0.432).

# Supplemental Figure 5. Forest plot of specificity according to study design.

**Supplemental Figure 5. Forest plot of pooled specificity according to study design.**
Forest plot showing the pooled specificity of AI-enabled ECG for the detection of acute myocardial infarction stratified by study design (retrospective vs prospective). Each square represents the study-specific estimate, with horizontal lines indicating 95% confidence intervals. Diamonds represent the pooled estimates for each subgroup and the overall analysis. Heterogeneity statistics (I²) and the test for subgroup differences are displayed.

# Supplemental Figure 6. Forest plot of pooled sensitivity according to study design.

**Supplemental Figure 6. Forest plot of pooled sensitivity according to study design.** Forest plot showing the pooled sensitivity of AI-enabled ECG for the detection of acute myocardial infarction stratified by study design (retrospective vs prospective). Each square represents the study-specific estimate, with horizontal lines indicating 95% confidence intervals. Diamonds represent the pooled estimates for each subgroup and for the overall analysis. Statistical heterogeneity (I²) and the test for subgroup differences are displayed.

# Supplemental Figure 7. Forest plot of pooled positive predictive value (PPV) according to study design.

**Supplemental Figure 7. Forest plot of pooled positive predictive value (PPV) according to study design.** Forest plot showing the pooled positive predictive value (PPV) of AI-enabled ECG for the detection of acute myocardial infarction stratified by study design (retrospective vs prospective). Each square represents the study-specific estimate, with horizontal lines indicating 95% confidence intervals. Diamonds represent the pooled estimates for each subgroup and for the overall analysis. Statistical heterogeneity (I²) and the test for subgroup differences are displayed.

# Supplemental Figure 8. Forest plot of pooled negative predictive value (NPV) according to study design.

**Supplemental Figure 8. Forest plot of pooled negative predictive value (NPV) according to study design.**
Forest plot showing the pooled negative predictive value (NPV) of AI-enabled ECG for the detection of acute myocardial infarction stratified by study design (retrospective vs prospective). Each square represents the study-specific estimate, with horizontal lines indicating 95% confidence intervals. Diamonds represent the pooled estimates for each subgroup and for the overall analysis. Statistical heterogeneity (I²) and the test for subgroup differences are displayed.

# Supplemental Figure 9. Summary receiver operating characteristic (SROC) curves according to study design.

**Supplemental Figure 9. Summary receiver operating characteristic (SROC) curves according to study design.** SROC curves illustrating the diagnostic performance of AI-enabled ECG for detection of acute myocardial infarction stratified by study design (prospective vs retrospective). Each circle represents an individual study. The solid curves represent the pooled SROC estimates for each subgroup, and the dashed ellipses indicate the corresponding 95% confidence regions around the summary points.

# References

[1]	Page MJ, McKenzie JE, Bossuyt PM, Boutron I, Hoffmann TC, Mulrow CD, et al. The PRISMA 2020 statement: an updated guideline for reporting systematic reviews. **BMJ** 2021;372:n71. <https://doi.org/10.1136/bmj.n71>.

[2]	Choi JWH, Torelli V, Silverman A, Diaz SS, Kong D, Vaish E, et al. AI-enhanced recognition of occlusions in acute coronary syndrome (AERO-ACS): A retrospective study. **Coron Artery Dis** 2025. <https://doi.org/10.1097/MCA.0000000000001555>.

[3]	Al-Zaiti S, Besomi L, Bouzid Z, Faramand Z, Frisch S, Martin-Gill C, et al. Machine learning-based prediction of acute coronary syndrome using only the pre-hospital 12-lead electrocardiogram. **Nat Commun** 2020;11. <https://doi.org/10.1038/s41467-020-17804-2>.

[4]	Chang KC, Hsieh PH, Wu MY, Wang YC, Wei JT, Shih ESC, et al. Usefulness of multi-labelling artificial intelligence in detecting rhythm disorders and acute ST-elevation myocardial infarction on 12-lead electrocardiogram. **Eur Heart J Digit Health** 2021;2:299–310. <https://doi.org/10.1093/ehjdh/ztab029>.

[5]	Alizadehsani R, Kragholm K, Barbagelata A, Chang KC, Wu MY, Wang YC, et al. Artificial intelligence-assisted remote detection of ST-elevation myocardial infarction using a mini-12-lead electrocardiogram device in prehospital ambulance care. **n.d.**

[6]	Riek NT, Akcakaya M, Bouzid Z, Gokhale T, Helman S, Kraevsky-Philips K, et al. ECG-SMART-NET: A Deep Learning Architecture for Precise ECG Diagnosis of Occlusion Myocardial Infarction. **n.d.**

[7]	Liu WC, Lin CS, Tsai CS, Tsao TP, Cheng CC, Liou JT, et al. A deep learning algorithm for detecting acute myocardial infarction. **EuroIntervention** 2021;17:765–73. <https://doi.org/10.4244/EIJ-D-20-01155>.

[8]	Gibson CM, Mehta S, Ceschim MRS, Frauenfelder A, Vieira D, Botelho R, et al. Evolution of single-lead ECG for STEMI detection using a deep learning approach. **Int J Cardiol** 2022;346:47–52. <https://doi.org/10.1016/j.ijcard.2021.11.039>.

[9]	Herman R, Mumma BE, Hoyne JD, Cooper BL, Johnson NP, Kisova T, et al. AI-Enabled ECG Analysis Improves Diagnostic Accuracy and Reduces False STEMI Activations. **JACC Cardiovasc Interv** 2026;19:145–56. <https://doi.org/10.1016/j.jcin.2025.10.018>.

[10]	Lee MS, Shin TG, Lee Y, Kim DH, Choi SH, Cho H, et al. Artificial intelligence applied to electrocardiogram to rule out acute myocardial infarction: the ROMIAE multicentre study. **Eur Heart J** 2025;46:1917–29. <https://doi.org/10.1093/eurheartj/ehaf004>.

[11]	Zhao Y, Xiong J, Hou Y, Zhu M, Lu Y, Xu Y, et al. Early detection of ST-segment elevated myocardial infarction by artificial intelligence with 12-lead electrocardiogram. **Int J Cardiol** 2020;317:223–30. <https://doi.org/10.1016/j.ijcard.2020.04.089>.
